# Nutrient recovery in cultured meat systems: Impacts on cost and sustainability metrics

**DOI:** 10.3389/fnut.2023.1151801

**Published:** 2023-04-06

**Authors:** Gabrielle M. Myers, Kate A. Jaros, Daniel S. Andersen, D. Raj Raman

**Affiliations:** Agricultural and Biological Systems Engineering, Iowa State University, Ames, IA, United States

**Keywords:** nitrogen, cultivated meat, *in vitro* meat, swine, broilers, beef

## Abstract

A growing global meat demand requires a decrease in the environmental impacts of meat production. Cultured meat (CM) can potentially address multiple challenges facing animal agriculture, including those related to animal welfare and environmental impacts, but existing cost analyses suggest it is hard for CM to match the relatively low costs of conventionally produced meat. This study analyzes literature reports to contextualize CM’s protein and calorie use efficiencies, comparing CM to animal meat products’ feed conversion ratios, areal productivities, and nitrogen management. Our analyses show that CM has greater protein and energy areal productivities than conventional meat products, and that waste nitrogen from spent media is critical to CM surpassing the nitrogen use efficiency of meat produced in swine and broiler land-applied manure systems. The CM nutrient management costs, arising from wastewater treatment and land application, are estimated to be more expensive than in conventional meat production. Overall, this study demonstrates that nitrogen management will be a key aspect of sustainability in CM production, as it is in conventional meat systems.

## 1. Introduction

Increases in global population and demographic changes, such as rising incomes and urbanization, are projected to increase the demand for animal-derived proteins in the coming decades (1). Relative to 2018–2020, total meat consumption is projected to increase by 14% by 2030 (2). To feed a population of over 9 billion in 2050, meat production is projected to increase by 58% compared to 2009 (3). Increasing the productivity of conventional meat production will be imperative to meeting humanity’s growing appetite (4). The animal production sector has already increased farm sizes and separated crop production from livestock production, for example, by shifting from pasture-based to confinement systems for animal production reliant on feed crops (5). These changes led to increased economies of scale, the development of optimized dietary feeds for animals, selective animal breeding programs, and specialized animal-rearing techniques, ultimately resulting in greater production efficiencies (6). Nevertheless, increasing total production has led to multiple negative environmental consequences (6–10). High-density animal husbandry raises consumer concerns about animal welfare (11) and amplifies manure handling difficulties (6, 9, 10).

A proposed approach to remedy these challenges is to use CM (also referred to as cultivated meat, cell-cultured meat, or *in vitro* meat) – a strategy proposed by Winston Churchill (12) involving the *in vitro* culturing of specific groups of animal cells for consumption by humans, thereby addressing animal welfare concerns (13). CM has been presented as a better alternative to conventional meat in terms of environmental impacts (14–16) and reducing the prevalence of antimicrobial resistance (17). Since the unveiling of the first CM burger in 2013 by Mark Post, over 100 CM production companies have been formed and are racing to bring CM to market across the globe (18). However, there are many challenges to overcome before CM is a viable large-scale alternative to conventional meat products (19). In CM production, high-cost culture media requirements and high capital investment have been identified as significant obstacles to market competitiveness (20, 21). CM companies face uphill battles in regulation and consumer acceptance (19, 22). While some believe these challenges to be unlikely to be overcome or the benefits to be questionable (23, 24), others point to rapid advancements and increasing investments made in the private sector and successful small-scale studies as a sign that CM will reach cost parity with conventional meat (14, 25–27).

In the U.S. Midwest region, crop and animal production are still intertwined, despite decades of decoupling the operations. Specifically, the staple crops corn and soybeans provide the nutrition needed at confinement operations, and animal manures provide valuable crop fertilizer (28). In Iowa - one of the largest producers of animal products in the US - 98% of cattle feedlot operations and 76% of swine farms apply their manure to cropland they own (28). Statewide, the manures can meet 30% of nitrogen demand and 50% of the demands of phosphorus and potassium in the state’s croplands (29). Reducing the volume of this manure through the use of CM production systems, although unlikely to fully replace animal production, may have implications for the circularity of agriculture in the region. A circular CM production system with waste media recycling has been proposed by Haraguchi et al. (30) with microalgae feedstocks (30). Other analyses have proposed the use of corn and soybean products in CM media as cost-effective energy and protein sources (15, 31, 32), and multiple studies present the potential for CM to reduce the global warming potential of meat products, especially when compared to beef (15, 16, 33). While some address eutrophication potential, they do not discuss if the remaining nutrients in the media are recycled to be applied to the land from which these products are harvested. In this work, we examine how nutrient cycling from CM in corn and soy fed system could affect its nitrogen use efficiency, compare the areal productivity of CM based on its protein and calorie use efficiencies to livestock land use, and discuss the costs of nutrient management.

## 2. Materials and methods

### 2.1. CM cost review

**FIGURE 1:**
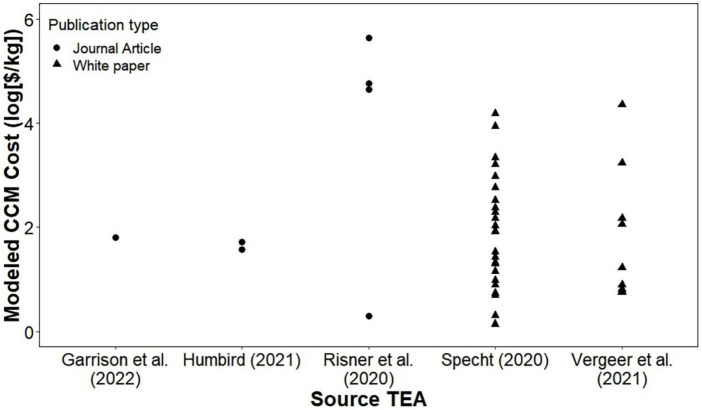
The cost estimates from five published technoeconomic analyses, distinguished by publication in a refereed journal or as a white paper. Log scale used to capture range of values.

Preliminary economic modeling efforts of CM production have been published in refereed and non-refereed outlets (14, 21, 32, 34, 35). Because industrial cell culture is primarily done in the high-value pharmaceutical industry, many current practices have not been optimized for cost or high production volumes as they would need to be in a food production scenario (36). Therefore, the results of published economic models vary widely based on their cell culture parameter assumptions and their cost projections for the future. Cell culture characteristics that differ between published results include maximum cell densities, reactor size, doubling time, feed requirements, and nutrient use efficiencies. These are critical assumptions in determining production volumes, costs, and media demands. Authors have projected costs of essential media components and capital expenditures if the market for CM-related feedstocks and equipment were to grow, with final CM costs ranging from over five orders of magnitude ($2 to $400,000 kg^–1^). This work uses published values in the comparisons made to traditional animal products. The range of TEA results is shown in Figure 1 – note the logarithmic scale, necessary to capture the extreme range of estimates. The Good Food Institute report authored by Specht (32) modeled only media costs, while the other analyses presented costs that included feed, capital, and labor. There is a large degree of communication between the Specht (32) model and the feed inputs assumed in the other models.

### 2.2. Growth parameters

To provide context for CM, we computed growth parameters for both CM and conventional meat products. The specific growth rate for all meat products (μ, units of day^–1^) was calculated per $\mu =\frac{l\,n\,\left(\frac{m_{f}}{m_{i}}\right)}{t}$, where *m*_*f*_ is the final mass, *m*_*i*_ is the initial mass, and *t* is the growth time (day). Because CM is assumed to be 100% edible, we only included the edible mass of traditional animal products in this calculation for better comparison. For CM, assumptions regarding batch size and length, cell mass, and cell densities needed to be made. As stated above, these values vary in published modeling efforts. Table 1 gives a summary of these assumptions presented in available models.

**TABLE 1:** A comparison of CM production assumptions applied or provided in several modeling efforts.

	References				
Variable	Risner et al. (21)	Humbird (31)	Mattick et al. (15)	Specht (32)	Tuomisto and Teixeira de Mattos (16)
Initial cell concentration* (cells ml^–1^)	1 × 10^5^–2 × 10^6^	8 × 10^6^**	2 × 10^5^	2 × 10^5^	–
Final cell concentration (cells ml^–1^)	1 × 10^7^–2 × 10^8^	3 × 10^7^**	4 × 10^6^	4 × 10^7^	1 × 10^7^
Proliferation phase length (h)*	53–160**	48*	118	240	–
Maturation phase length (h)	24–240	–	72	240	1,440***
Final mass per cell, wet (g)	3.5 × 10^–9^	3 × 10^–9^	3.5 × 10^–9^	4.4 × 10^–9^**	3.3 × 10^–9^
Final reactor size (m^3^)	20	20	15	20	1

*In the final bioreactor, i.e., not including seed bioreactors.

**Calculated from parameters given in the paper; not explicitly stated by authors.

***Phases are not separated, but 60 days given for total batch time.

The parameters used by Mattick et al. (15) align with the other published analyses listed in Table 1, so these were chosen to compare to animal products (15). The final CM mass was calculated by multiplying the end of final cell density by the final mass of a cell and the reactor volume, effectively assuming that all reactor volume is working volume and all the biomass can be harvested. The CM produced is assumed to have a 70% moisture content (31) and 100% edible tissue. An estimated total feed requirement was calculated as the mass of protein and glucose required to feed one batch of CM (discussed further below). This does not include other critical media components, such as essential vitamins, pH control ingredients, and growth factors. Some of these ingredients may be recycled between batches as they would not be fully consumed by the growing cells, but ingredient recycling technology requires further development to be cost-effective (31, 37).

For the conventional animal products, we collected literature data on birth weight, live weight, edible meat percentage, meat output (wet basis), meat protein content, meat moisture content, growth time, average daily weight gain, specific growth rate, feed conversion ratio, total feed mass, protein in the diet, protein fed, and protein yield for each animal protein source. Animal birth weights were determined from published reports (38–40). The traditional animal products’ feed conversion ratios, protein content in the feed, and edible meat percentages were gathered (41). To calculate the meat yield of each animal, we multiplied the edible meat percentage by the slaughter weight (42–44). When evaluating meat protein and moisture content, we used reported nutritional values for ground meat for all traditional animal protein sources (45–47). The broiler growth period was reported by the National Chicken Council (42). Swine and beef growth periods were based on estimates of the typical growth time required for animals (48). We calculated the average weight gain in kg day^–1^ by taking the live weight minus the birth weight and dividing it by the growth period. We calculated the total feed intake by multiplying the slaughter weights of animals by their respective feed conversion ratio. The protein input for animals was calculated by multiplying the feed protein content by the total feed intake. Multiplying the meat output by the meat’s protein content allows us to determine the protein output of meat. To determine the protein conversion efficiency (PCE), we divided the protein output of the meat by the protein input. The gestation period of the animals was not included in this analysis.

### 2.3. Areal productivity

Areal productivity represents the amount of protein or energy produced from a given land area. To compare the areal productivity of CM to existing protein and calorie sources, a range of land use values for conventional meat products were obtained from reviews of life cycle assessments (49, 50). Most of the life cycle assessment studies contained in the reviews focused on European or North American production systems. Median land use values for extensive (grazing), intermediate, and intensive (feedlot) beef production were converted to energy and protein areal productivities using the energy and protein contents of 97% lean ground beef (47). Beef land use values ranged from 15 to 429 m^2^ kg^–1^ year^–1^ (49). The land use median for broiler production of 8.7 m^2^ kg^–1^ year^–1^ (50) was converted to protein and energy productivities, assuming 1,430 kcal (6,000 kJ) kg^–1^ chicken and 17% protein (46). Beef and broiler land use numbers were reported as ranges, so midpoint averages were used. Swine land use was between 8 and 15 m^2^ kg^–1^ year^–1^, so a midpoint value of 11.5 m^2^ was used (49). This was converted to energy and protein productivities assuming 2,630 kcal (11,000 kJ) kg^–1^ and 17% protein content (45).

The areal productivity of CM will vary widely based on the feed inputs assumed. Previous CM life cycle analyses present land use values ranging from 0.2 m^2^ kg^–1^ (16) to 5.5 m^2^ kg^–1^ (15). Our analysis is comprised of the feed inputs of corn and soybeans to supply the necessary amino acids and glucose according to the energy and PCE. We did not consider the land required for the CM production facility, but the Mattick et al. (15) land use analysis showed this was small compared to the land required for agricultural production [98% of total land use; (15)]. This analysis does not consider the addition of cultured adipose tissue. Energy and protein conversion efficiencies of 17 and 24%, respectively (51), were used as the basis of CM land use requirement computations. Protein was assumed to be sourced from soybean hydrolysate (31), despite it missing the required amino acid glutamine (31, 52, 53); no correction was made for this deficit. Soybeans were portioned to approximately 20% oil and 80% meal. The protein content of soybean meal was assumed to be 48%, with 80% of this protein recoverable in the hydrolysate (31). The average yield of soybeans in Iowa in 2021 was 4.12 Mg ha^–1^ [62 bu acre^–1^; (54)], which is equivalent to a land use of 2.4 m^2^ kg^–1^. This land use requirement was adjusted to account for the soybean oil portion of the soybean yield. This adjustment was made based on value. The 2019–2021 average soybean oil price is $0.87 kg^–1^ (55), and the 2019–2021 average soybean meal price is $0.33 kg^–1^ (56). After adjusting the values based on their presence in soybeans, oil was assigned 39% of the land use for soybean production, and meal was allocated 61%, which was then applied to CM production. The wet CM protein content was assumed to be 18% (31). Using these parameters, we calculated a land use requirement for supplying protein to CM.

The energy input requirement to CM was assumed to be sourced from glucose in corn. Assuming 4,000 kcal kg^–1^ glucose, the fed requirements of glucose were calculated to be approximately 2 kg glucose per kg of meat produced using a 17% caloric conversion efficiency (51) and a calorie content of 1.4 kcal g^–1^ CCM (31). This value is considerably lower than the 26–33% calorie conversion efficiency presented as possible in Humbird (31), so it could be considered conservative (31). In the wet milling process, 0.67 kg of starch is obtained from each kg of corn (57). The average yield of corn in Iowa was 13 Mg ha^–1^ in 2021 [205 bu ac^–1^; (54)] for a land use requirement of 0.78 m^2^ kg^–1^. Again, this land requirement was partitioned between the corn starch used for CM production and the remaining corn gluten meal based on their values. Price-based allocation analysis by Mattick et al. (15) showed that approximately 74% of the impact from corn should be allocated to corn starch, which was applied to our CM land use calculation (15). It is worth noting that this analysis neglects the non-feed energy inputs required in CM production. Maintaining reactors at the proper temperature, cleaning, mixing, filtration of waste products, and sterilization will likely require much higher direct energy inputs to the system than are required in conventional meat production (51). After the land requirements for the energy and protein inputs were established, they were summed, and protein and energy areal productivities for CM were calculated.

### 2.4. Spent media nitrogen management

Media recycling is mentioned as a cost and material-saving strategy in several CM publications (19, 30, 32, 33, 37), noting that recycling will depend on media composition and available technology. Previous research has highlighted the need for media recycling to lower feed costs, particularly regarding expensive ingredients such as growth factors and hormones (32). However, focusing on these high-cost ingredients overlooks the treatment of other parts of the spent media, and studies rarely discuss the impact of nutrient conversion efficiencies on the advertised benefits of the system. In traditional animal agriculture, the value of nitrogen in manure is recognized, so the manure is applied to the crops that provide the main feed for the animals, reducing the reliance on synthetic fertilizers and creating a circular nutrient system.

Several factors may impact nitrogen reuse in a CM production system, including location, scale, and final concentration. The location of the production site may depend on scale, with a large-scale system potentially benefiting from being located near the production of its main media ingredients. Additionally, if CM is to have a similar level of circularity to conventional meat, the economic land application of the nutrients in the spent media may require proximity to croplands. The final nitrogen concentration in the spent media depends on the volume of water needed, the PCE, and the final cell concentrations. As discussed above, these values vary in published models and may not be fully known until the publication of industry recipes.

For this analysis, we use a mass balance approach to calculate the final nitrogen concentration in the spent media based on the assumed PCE of 24% (51). Mattick et al. (15) propose a system that utilizes approximately 30 m^3^ of water for the production of 345 kg CM in a 15 m^3^ reactor. It is worth noting that this water volume does not account for the cleaning of the reactors [an additional 45 m^3^ per batch in Mattick et al. (15)], which we assume is handled separately from the spent media stream. Assuming the meat’s protein content to be 18%, the PCE indicates a feed requirement of 260 kg protein per batch, of which 62 kg will become protein in the meat. This leaves approximately 200 kg of protein in the spent media, or 31 kg of nitrogen [16% nitrogen content in protein (58)]. We are assuming that even a small-scale CM facility would not operate with a revenue lower than $10 million per year due to the high level of skilled labor and capital investment required. This assumed revenue requirement may be optimistic, as the modeled annual capital expenses alone in Humbird (34) approach $50 M (34). If we assume a relatively high price for a ground meat product of $25 kg^–1^, a single facility would need to produce 400,000 kg CM. In production scenario presented by Mattick et al. (15), this meat mass equates to 1,160 batches annually. A price of $10 per kg, which is more in line with the price of traditional ground meat, would require the production of 1,000,000 kg CM, or 2,900 batches. Note that the batch time presented by Mattick et al. (15) is 11 days. This batch time corresponds to a maximum of 33 batches per reactor annually. These high-price and low-price scenarios would require 35 and 88 reactors, respectively.

The high-price, low-production scenario results in a waste stream of 36 Mg N, while the low-price, high-production scenario results in 91 Mg. The meat in the Mattick et al. (15) model has an 83% moisture content, so approximately 1% of the water input ends up in the meat. The wastewater volume is the difference between the volume inputted and the volume in the meat and equals 34.4 × 10^3^ m^3^ in the high-price scenario and 86.1 × 10^3^ m^3^ in the low-price scenario. In each case, while the total mass of nitrogen differs significantly, the spent media nitrogen concentration is 1.06 kg N m^–3^. (1,060 mg L^–1^) In reality, the concentration could be much lower if the water required for cleaning or the spent media from earlier production stages were included in the same waste stream. This concentration is lower than the concentration of N typical of livestock manures (48).

We first considered the cost of land-applying all of the waste stream in its present concentration. For this calculation, we assumed that the CM plant was located in the center of the cropland to which it would be applying nitrogen and that nitrogen would be applied at a rate of 168 kg ha^–1^(150 lbs N acre^–1^). In the high meat price (low meat production) scenario, the land requirement would be 217 ha, while it would be 543 ha in the higher production, low-price scenario. In 2021, 35% of Iowa’s land was growing corn (59). Therefore, we applied this percentage to the calculated land area to determine the total distance that the spent media would need to be transported. This is likely an underestimation of the cropland that would surround a CM facility land applying spent media, as producers would choose a location more surrounded by corn-growing cropland. This adjustment raises the land application area requirement to 620 ha in the high-cost scenario and 1,550 ha in the low-cost scenario. These areas can be conceived as circles with radii of 1.4 km (0.87 miles) and 2.2 km (1.38 miles), respectively. Several sources place liquid manure application costs at approximately $0.01–0.015 gallon^–1^ (60–62), with Andersen noting that when analyzing a commercial applicator rates survey, cost also increased for each mile the manure was hauled. A baseline cost of $0.0125 gallon^–1^ was assumed. In the high-cost scenario, the spent media can all be applied within a one-mile radius of the CM production facility. In the low-cost scenario, 48% of the land area would be greater than one mile away, incurring an additional $0.0035 gallon^–1^ cost (61).

The cost of treating all of the spent media for nitrogen in both meat cost scenarios was calculated as $2.45 kg^–1^ N ($1.11 lbs^–1^), which is the cost of treating Total Kjeldahl Nitrogen (TKN) in Ames, Iowa (63). The nitrogen waste mass flow calculated for both cost scenarios above was applied here. In the wastewater treatment cost calculation, we also considered the cost of treating carbonaceous waste (as COD – Chemical Oxygen Demand), which costs $0.40 kg^–1^ COD ($0.18 lbs^–1^) in Ames, Iowa (63). For the COD treatment cost, we had to consider the fate of the fed carbon. From the caloric conversion efficiency, we assumed that 17% of the fed glucose became glucose in the meat. We utilized the oxygen uptake rate in the Mattick model (332.2 nmol O_2_ h^–1^ 10^6^ cells^–1^) to determine the respiration rate (15). We assumed a median cell count during the proliferation phase (118 h) from the model’s given initial and final cell densities, and we applied the final cell count to the entirety of the differentiation phase (72 h). This resulted in 72 kg of oxygen uptake per batch, requiring 80 kg of glucose to be respired. From the 2 kg glucose per kg CM feed requirement calculated in the areal productivity section, we can calculate a total glucose input of 710 kg per Mattick batch. Of this feed input, 11.3% is respired, 17% is retained in the meat, and the rest is considered waste. The mass of glucose waste was converted to carbon mass for both cost scenarios (0.4 kg C kg^–1^ glucose). A conversion factor of 2.66 COD kg^–1^ carbon in glucose was used to convert carbon waste to COD (64).

We also wanted to compare the costs of waste management between animals and CM, as this has been absent from previous analyses. To calculate waste management costs for livestock, we first calculated total manure production for beef, broilers, and swine according to ASAE Standard D384.2 (48). For beef, the finished animal manure production for finishing cattle was added to the daily manure production for a growing calf multiplied by the typical length of this stage (240 days). The lifetime calculated manure production for beef was 9,800 L animal^–1^. For swine, the finished animal manure production for nursery pigs and grow-finish pigs was added, which resulted in 600 L animal^–1^. For broilers, the single number for finished animal manure production was used (5 L animal^–1^). A manure application cost of $0.0125 gallon^–1^ was applied as above.

Finally, we wished to provide a comparison between nitrogen conversion efficiencies that includes recoverability. To compute the values for conventional meat products, we used published values for N available in manure (29), weighted averaged over multiple life stages (48), and corrected with a volatilization factor of 0.98 for direct injection (65). We then assumed a 20% leaching loss. Protein fed to the animals, as calculated previously, was converted to nitrogen mass. The recoverable nitrogen percentage was calculated as the difference between the available nitrogen for application after volatilization and the leaching loss divided by the nitrogen fed to the animal. The total loss was assumed to be the remaining percentage after the recoverable nitrogen and the PCE. This calculation will provide a basis for how much nitrogen would need to be recovered in the CM production system to be as circular as conventional meat production systems.

Because application costs are based on both distance and volume, a more concentrated nitrogen solution is less costly to apply. Wastewater treatment strategies can recover up to 75% of nitrogen mass into a more concentrated stream (66). The more concentrated nitrogen solution could be applied to cropland, while the rest of the spent media would be sent to a wastewater treatment facility. With the price of nitrogen as a fertilizer between $1–3 kg^–1^ (67), the cost of this recovery would need to be well below that. This work does not explore the costs of deploying this strategy, but it could reduce application costs of any scale CM production and be especially useful if cleaning water is included in the waste nitrogen stream.

## 3. Results

### 3.1. Growth characteristics

The growth characteristics of CM, swine, beef, and broilers are shown in Table 2.

**TABLE 2:** Growth and feed use characteristics of beef, swine, and broilers compared to cultured meat.

Quantity (units)	Swine	Beef	Broiler	CM
Birth weight* (kg)	1.4	35	0.04	10.5
Live weight* (kg)	130	600	2.8	345
Edible meat (%)	52%	40%	46%	100%
Meat output wb (kg)	66	240	1.3	345
Meat protein content (kg protein/kg meat_wb_)	0.17	0.22	0.17	0.18
Meat moisture content (%)	61%	75%	73%	70%
Growth time (day)	180	640	47	8
Average weight gain (kg/day)	0.70	0.88	0.06	42
Specific growth rate (day^–1^)	0.025	0.004	0.091	0.61
Feed conversion ratio (kg feed/kg L.W.)	3.1	14	1.9	2.8
Total feed mass (kg feed)	400	8,400	5.40	970
Protein in diet (%)	17%	12%	17%	27%
Protein fed (kg)	68	1,000	0.92	259
Protein conversion efficiency	17%	5.0%	25%	24%

*For cultured meat, these refer to the inoculum and masses in the final production mass.

### 3.2. Areal productivity

**FIGURE 2:**
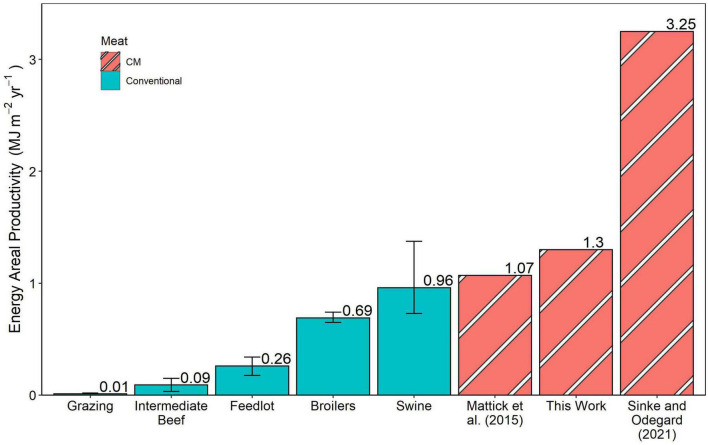
Energy areal productivity in this calculated in this work, calculated from published land uses in CM life cycle analyses, and from published land uses of conventional meat products shown in MJ m^– 2^ year^– 1^. Error bars represent the range of published land use values in conventional meat production.

**FIGURE 3:**
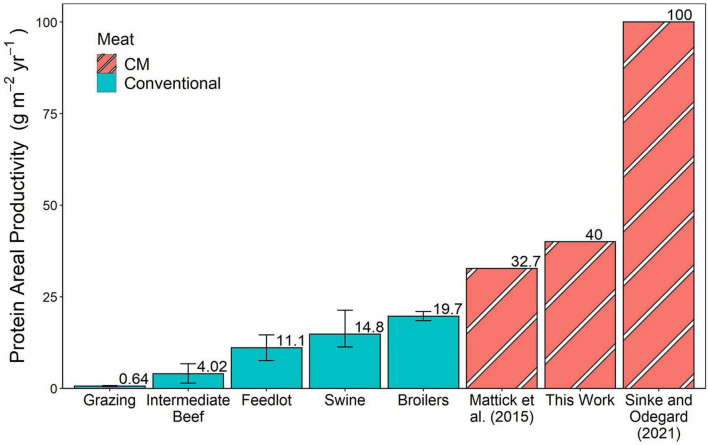
Protein areal productivity in this calculated in this work, calculated from published land uses in CM life cycle analyses, and from published land uses of conventional meat products shown in g protein m^– 2^ year^– 1^. Error bars represent the range of published land use values in conventional meat production.

After applying the price-based allocation method described above, the land used to reach the protein requirement through soybeans was 2.31 m^2^ kg^–1^ CM. Meeting the calorie requirements through corn required 2.28 m^2^ kg^–1^ CM. Summing these results in a 4.58 m^2^ kg^–1^ CM is required. This value translates to 40 g protein and 1.3 MJ produced per square meter under our assumed CM protein and calorie contents. Land use results from Mattick et al. (15) and Sinke and Odegard (33) life cycle assessments were similarly converted to areal productivities (15, 33). Figures 2, 3 show these energy and protein areal productivity results, respectively, compared to those found from converting published animal land use values to areal productivities. The error bars in conventional meat production represent the range of land use values obtained from the life cycle analysis reviews.

### 3.3. Spent media nitrogen management

The annual costs of land-applying spent media and treating spent media for nitrogen and COD, and the associated calculations for each are outlined in Table 3. For both scenarios, the cost of wastewater treatment is approximately $0.85 kg^–1^, with $0.62 attributed to the cost of treating COD and $0.22 attributed to the cost of treating nitrogen. The specific cost of applying the spent media is $0.28 kg^–1^ CM in the high-cost scenario and $0.32 kg^–1^ in the low-cost scenario.

**TABLE 3:** Spent media nitrogen and COD production and management costs for two CM production scenarios.

	High-cost CM ($25 kg^–1^)	Low-cost CM ($10 kg^–1^)
Annual CM production (kg)	400,000	1,000,000
Waste nitrogen (kg year^–1^)	36,500	91,200
Waste COD (kg year^–1^)	628,000	1,570,000
Wastewater treatment Cost ($ year^–1^)	339,000	847,000
Application cost ($ year^–1^)	114,000	332,000

### 3.4. Livestock waste handling comparisons

**FIGURE 4:**
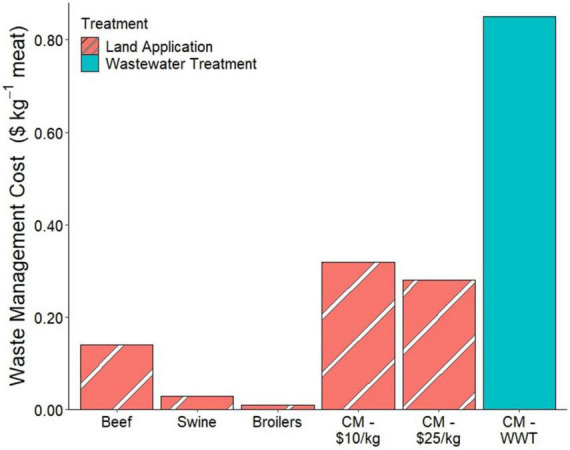
Costs of nutrient management strategies for conventional meat production, two CM scenarios for land application, and CM wastewater treatment (WWT) for COD and nitrogen.

Figure 4 shows the cost of land application of manure for beef, swine, and broilers, compared to the cost of land applying spent media for CM in the two production scenarios discussed and the cost of wastewater treatment of the CM spent media. These calculations showed nutrient management in CM to be more expensive than in current meat production systems. The lower-cost CM scenario results in greater meat production and a larger area for spent media to be applied, resulting in the higher cost of land application. The wastewater treatment cost is higher than the land application cost, but wastewater treatment would be the only option if CM production facilities were not located close to the croplands that supply their feed components.

**FIGURE 5:**
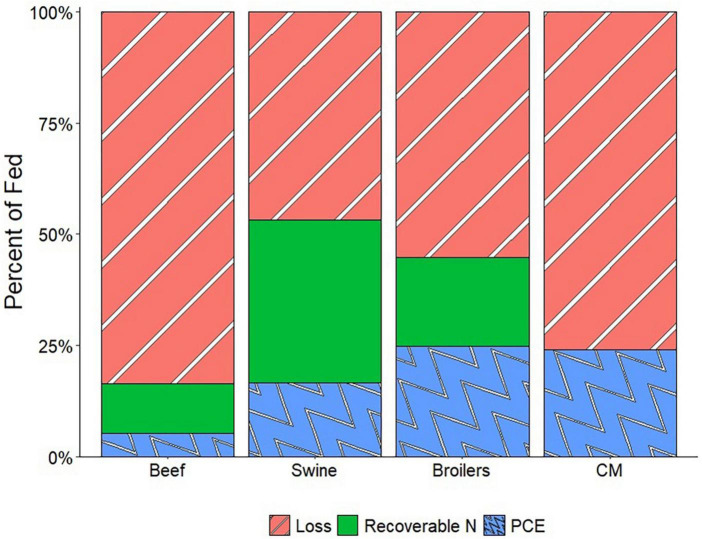
Nitrogen use efficiencies compared for beef, swine, broilers, and cultured meat with no nitrogen recovery. Values are based on protein conversion efficiencies (PCE) and nitrogen availability in livestock manures.

Figure 5 shows the results of the nitrogen use efficiency comparison between animals and CM. Without nitrogen reuse or recovery, CM production would result in 76% of nitrogen fed as waste, compared to 84% for beef, 47% for swine, and 55% for broilers.

## 4. Discussion

### 4.1. Growth parameters

Cultured meat is projected to have a much higher specific growth rate than swine, beef, and broilers. In some sense, this high specific growth rate underlies the capacity of CM systems to pay the significant capital costs associated with such a technologically intensive production system. However, the feed conversion ratio of broilers is lower than in CM, and swine is not far behind. This suggests that more feed mass is required to produce a kilogram of CM than for broilers to gain one kilogram of weight. Beef cattle were estimated to have the highest feed conversion efficiency and the lowest PCE, which suggests that CM should target beef products if the industry aims to make the most significant impact.

### 4.2. Areal productivity

Figures 2, 3 show that multiple CM estimations’ energy and protein efficiencies surpass those of conventional meat products. We model CM beef production, which surpasses feedlot beef areal energy productivity by a factor of 5 and protein by a factor of 3.6. The CM beef exceeds the best median conventional meat systems by 30 and 100% on these metrics, respectively. However, the best case swine land use value is more productive for energy than two of the CM estimations. Overall, these results suggest that CM could lead to decreased land footprints for meat production. This calculation was completed assuming CM feed would get its protein and calorie requirements from corn and soybeans. In industrialized animal meat production, a large portion of the environmental impact is attributed to the growth of corn feed (68) and other feed inputs may reduce land use requirements. It is currently unclear from what crops these portions of the feed will be derived in practice, and different sources will have different areal productivity implications.

While grazing beef cattle is the least efficient land user identified, a critical caveat is that grazing livestock – in contrast to feedlot-raised livestock and CM – harvest calories and protein from land that is typically not favorable to producing row crops like corn and soybean, which are required for meat substitutes. One can imagine a future in which a combination of plant-based meat substitutes and CM provides a large share of what is now provided by conventional (including feedlot) agricultural operations, while meat from grazing livestock serves a smaller market. Additionally, while other analyses point to the possibility of using soybean hydrolysates and corn sugar for providing protein and calories to CM production, other feedstocks will likely be needed to supplement the media to meet all amino acid requirements.

Our soybean requirement analysis is more conservative than Humbird (31), who presented the stoichiometric requirement to meet the amino acid requirements (excluding glutamine) of 0.33 kg of soybean hydrolysate per kg of meat. In contrast, the analysis detailed above results in 0.75 kg soybean hydrolysate required per kg of meat because it accounts for conversion losses. Land use in (15) is 5.5 m^2^ kg^–1^ CM, which is greater than our calculated land use. Our land use only includes the land use for soybean hydrolysate and glucose from corn inputs, while the Mattick paper includes others. In contrast, the land use in (33) is 1.7–1.8 m^2^ kg^–1^ CM, 0.19–0.23 m^2^ kg^–1^ CM in (16), and 0.39–0.77 m^2^ kg^–1^ CM in (69). Differences in feedstock inputs and ambitious media recipes/use lead to these differences. Still, in each case, the productivity of CM is projected to be greater than that of its animal meat counterparts.

### 4.3. Spent media and nitrogen handling

Nitrogen cycling is an important part of traditional meat production systems. The plant-available nitrogen in animal manures provides fertilizer for the crops used as their feed. The cost of waste management in CM production calculated in this paper is low compared to the overall prices modeled for CM (Figure 1). Because of this, in the push to get CM to be cost-competitive with traditional meat products, nitrogen recovery is likely to be overlooked while producers focus on the recovery and reuse of expensive portions of the media. Throughout history, one of the primary drivers of improved nutrient use efficiency in meat production was incremental improvements in feed conversion efficiency. However, as shown in Figure 5, CM production without nitrogen recovery is less efficient in nitrogen use than swine and broilers. Moreover, the integration of crop-livestock production systems is typically suggested as a means to achieve greater sustainability. To be as efficient in nitrogen use as broilers, nitrogen would need to be recovered at a rate of 21% of the fed nitrogen (27% of the waste stream). To be as efficient in nitrogen use as swine, nitrogen would need to be recovered at a rate of 29% of the fed nitrogen (38% of the waste). The cost of the land application in the low and high CM cost scenarios was $0.32 and $0.28 kg^–1^ CM, respectively. These costs are 2–10× greater than the calculated animal manure land application values because the spent media is more dilute in nitrogen than typical animal manures. However, the land application cost is still much less than the price of nitrogen fertilizer (67) and would therefore be valuable to crop farmers.

A large-scale CM production system using a wastewater treatment approach to nutrient management rather than a storage and land application approach consistent with current livestock production systems is a drastic change in waste management strategies. In terms of costs, this strategy amounts to significantly higher monetary investment than current meat production strategies incur. This strategy is likely for production facilities located far from croplands or with very dilute waste streams. If CM production were to reach large scales and displace livestock production, this could cause a nitrogen imbalance in the landscape.

The high and low-cost CM production scenarios calculated would create 36 and 91 Mg of waste N annually. For context, a human nitrogen excretion rate is approximately 13 g person^–1^ day^–1^ (58). The high-cost scenario is 7,700 person-equivalents (PE) in nitrogen waste, while the high-cost scenario is 19,000 PE. These numbers are similar to those of small cities and would likely place a major strain on local wastewater treatment plants unless situated in very large population areas. With this high nitrogen production, the highly trained professional labor at a CM plant, and the availability of capital, these facilities might regularly deploy sophisticated nutrient recovery systems far beyond what has been viable in the animal production industry. These approaches could yield concentrated fertilizer streams with low moisture contents – such streams could be economically transported long distances and might enable a further decoupling of meat production and crop production, which may have profound impacts on rural states that currently benefit economically from animal production systems co-located with crops.

## 5. Conclusion

Increased nutrient use efficiencies have been an advertised benefit of CM production. Nutrient management is a critical aspect of the sustainability of current meat production systems, and waste management has been largely left out of previous CM analyses. This study found that the energy and protein areal productivity of CM is likely higher than traditional meat products based on published calorie and protein conversion efficiencies and land use values published in life cycle analyses. However, CM may be less efficient than conventional meat in terms of nitrogen use if the nitrogen is not recycled or applied to cropland as in traditional meat production systems. Spent CM media handling was estimated to be costlier than manure applications. Future research could provide more information on CM nutrient conversion efficiencies and spent media nutrient concentrations to more fully understand the nutrient cycling implications of CM displacing animal meats.

## Data availability statement

Publicly available datasets were analyzed in this study. This data can be found here: https://github.com/gabbymyers/Cultured-Meat-Nitrogen-Cycling.

## Author contributions

DA and DR conceived of the analysis and edited the manuscript. GM and KJ completed the necessary analyses. GM prepared the original draft of the manuscript. All authors contributed to the article and approved the submitted version.
